# Educational Pain Points for Pediatric Brain Tumor Survivors: Review of Risks and Remedies

**DOI:** 10.3390/children8121125

**Published:** 2021-12-03

**Authors:** Peter L. Stavinoha, Thuy Trinh-Wong, Laura N. Rodriguez, Chawncey M. Stewart, Kris Frost

**Affiliations:** Division of Pediatrics, University of Texas MD Anderson Cancer Center, Houston, TX 77030, USA; TTTrinh1@mdanderson.org (T.T.-W.); LNRodriguez@mdanderson.org (L.N.R.); CMStewart1@mdanderson.org (C.M.S.); MKFrost@mdanderson.org (K.F.)

**Keywords:** brain tumor, pediatrics, education, psychosocial, school, quality of life

## Abstract

Evolving treatment paradigms have led to increased survival rates for children diagnosed with a brain tumor, and this has increasingly shifted clinical and research focus to morbidity and quality of life among survivors. Among unfavorable outcomes, survivors of pediatric brain tumors are at risk for academic failure and low educational attainment, which may then contribute to lower health related quality of life, lower income and vocational status, and a greater likelihood of dependence on others in adulthood. Several specific risk factors for lower educational performance and attainment have been investigated. These are typically examined in isolation from one another which clouds understanding of the full range and potential interplay of contributors to educational difficulties. This review integrates and summarizes what is known about the direct and indirect barriers to educational success and performance (i.e., educational pain points) to enhance clinician knowledge of factors to consider when working with pediatric brain tumor survivors. Specific barriers to educational success include neurocognitive difficulties, school absences, psychosocial challenges, challenges to knowledge and communication, and physical and sensory difficulties. Finally, we discuss the current state of educational interventions and supports and offer recommendations for future research to improve educational outcomes for pediatric brain tumor survivors.

## 1. Introduction

Brain tumors comprise more than 15% of pediatric cancers and are the second most common cancer in children and adolescents [1]. While advances in treatment have improved survival rates, a substantial body of literature documents potential late effects (i.e., delayed emergence of psychosocial, neurocognitive, and health-related sequelae) experienced by pediatric brain tumor (PBT) survivors [2]. PBT survivors tend to have the lowest health related quality of life (HRQOL) among all pediatric cancer types [3], and this population is at significant risk for dependence on others into adulthood [4]. Functioning at school is a component of HRQOL and is consistently cited as problematic for PBT survivors [5]. Multiple factors and late effects contribute to educational difficulties which represent significant pain points for this population [2].

PBT survivors consistently perform below healthy peers and other pediatric cancer survivors across metrics of educational performance. PBT survivors are more likely to fail subjects at school [6], repeat a grade [7,8], perform more poorly than peers on standardized tests [9,10], and require more educational support [11,12] relative to healthy peers and other pediatric cancer survivors. Thus, PBT survivors experience lower educational attainment compared to healthy peers and other cancer survivors [13]. Subsequently, the PBT population experiences additional pain points as they transition to adulthood, with lower incomes and higher rates of unemployment compared to healthy peers and other pediatric cancer survivors [14,15]. Indeed, the risk for unemployment increases with lower levels of educational attainment [15]. Finally, factors that are difficult to change, such as socioeconomic status (SES) can be both a protective factor and risk factor, with evidence suggesting that higher SES is protective of cognitive and academic functioning whereas lower SES is associated with worse cognitive and educational outcomes [16].

In short, educational performance and attainment are clear barriers to optimal independence and HRQOL for PBT survivors. Educational pain points for PBT survivors are well-documented, and a constellation of factors contributes to decreased educational performance and attainment. However, the literature tends to focus on one aspect or another without consideration of the full range, and potential cumulative effects, of risk factors for educational difficulties in PBT survivors. Thus, our goal is to summarize direct and indirect challenges to educational success for pediatric brain tumor survivors. This includes a review of factors, such as neurocognitive difficulties, school absences, psychosocial challenges, challenges to knowledge and communication, and physical and sensory difficulties. Finally, the current state of educational interventions and supports is discussed and recommendations for future research are made to integrate educational performance and outcomes into therapeutic trials for PBT survivors.

## 2. Educational Pain Points for Pediatric Brain Tumor Survivors

### 2.1. Neurocognitive Variables

A large body of literature documents neurocognitive late effects of pediatric brain tumors and treatment [2,17]. Late effects include tumor and treatment related cognitive changes that typically emerge in the first few years following treatment and may range from little or mild change to severe deficits that require the ongoing need for significant support into and through adulthood [2]. Unlike other neurological conditions that may affect cognition acutely, such as traumatic brain injury or stroke, the trajectory of impairments for brain tumor survivors can be more protracted, with deficits becoming apparent sometimes years after treatment has ended.

Numerous risk factors related to late neurocognitive effects have been documented. Tumor size [18], tumor location [19] and potential for complications, such as obstructive hydrocephalus affect neurocognitive outcomes independent of the treatment paradigm [20]. Younger age at diagnosis, longer time since treatment, genetic predispositions, chronic health conditions including ototoxicity, endocrinopathies, cardiac disease and other complications, such as epilepsy or stroke may increase the risk for, and the manifestation of neurocognitive late effects as time goes by [17].

Essential treatment modalities also pose risk to neurocognitive functions, thereby impacting educational performance and attainment. Cranial radiation therapy (CRT) is often identified as the most impactful and deleterious treatment-related risk factor to neurocognitive outcomes for PBT survivors due to white matter changes that associate with neurocognitive dysfunction and persisting late effects [21]. Chemotherapy, while thought to be less toxic relative to radiation treatment, has been associated with cognitive impairment [22,23], as well as hearing impairments [24] thus, magnifying the functional impact of neurocognitive late effects. Independent of other treatment modalities, neurosurgical resection of tumors is associated with neurocognitive morbidity [25,26] and in some cases results in the post-surgical complication of posterior fossa syndrome (also known as cerebellar mutism) characterized by diminished speech, ataxia, emotional and behavior lability, and apathy [27] which may lead to worse long term neurocognitive outcomes for PBT survivors [28].

Historically, overall IQ has been a frequently cited vulnerability following treatment for PBT [21,29,30]. Recent evidence suggests that these overall declines may be better accounted for by specific cognitive abilities that both support overall intellectual ability and are at risk due to PBT, treatment, and associated complications [31]. For example, core deficits most commonly reported following treatment for PBT include attention, working memory, and processing speed, functions which may decline over time [21,32,33,34,35]. 

Deficits in executive functions, a broad cluster of cognitive functions related to purposeful, goal-oriented, problem-solving behaviors and self-regulation, are also at significant risk in PBT survivors across ages, tumor types, and treatment regimens [36,37,38,39]. Within the classroom setting, teachers rate PBT survivors as experiencing rates of clinical impairment in executive functions ranging from 3–10 times that of typically developing peers [40]. 

While neurocognitive late effects clearly affect a significant number of PBT survivors, a relatively consistent finding has been that PBT survivors generally demonstrate broadly average performances on standardized measures of basic academic skills [41,42,43]. This is particularly perplexing in light of the clearly and consistently documented difficulties with educational performance and attainment described above. However, these measures of basic skills may not reflect the real-world educational demands on the PBT survivor which require significant coordination of neurocognitive functions in addition to intact discrete neurocognitive functions [41]. Further, this pattern indicates that other factors, beyond neurocognitive dysfunction, contribute to educational pain points experienced by PBT survivors.

### 2.2. School Absences and Limited School Engagement

It is no surprise that school absenteeism is associated with academic underachievement, low educational attainment, and increased risk for unemployment [44]. Beyond missed educational opportunities, multiple additional risks highlight the importance of improving our understanding of reasons for school absences in PBT survivors and the resulting range of negative impacts. Better recognition of these nuanced and often interrelated factors may lead to improved efforts to ameliorate school absences and their impact on PBT survivor educational success.

School absences are especially problematic during the first-year post-diagnosis when the child is in the acute stages of treatment and recovery [45,46]. Even long after treatment is complete, PBT survivors continue to experience a higher rate of school absences relative to healthy peers as well as relative to other pediatric cancer types [47], with more absences associated with lower achievement [48]. In addition to medical care, reasons cited for school absences include physical illness, fatigue, and parental concern [48].

Limited school engagement is a deleterious byproduct of absenteeism that is only recently getting attention in the PBT survivor literature. School engagement involves cognitive investment and effort, participation in social academic and extracurricular activities, and emotional influences on willingness to do the work of school [49]. Engagement in school and school-related activities, such as sports, languages, and play is more limited in PBT survivors [50,51], and this may have negative implications for later educational and occupational success [52].

Beyond the obvious connection between school attendance and availability to benefit from instruction, absenteeism can negatively affect the development and maintenance of social interactions and relationships and can result in fewer friendships at school [48,53,54]. Further, PBT survivors are at greater risk of peer victimization and bullying at school [48] and have cited fear of peer rejection as a reason for school absences [45], factors which can lead to school phobia and school refusal [55].

Thus, school avoidance may be a factor for some PBT survivors as a manifestation of anxiety, social incompetence and fewer friendships, and difficulty with self-esteem that survivors experience [47,48,56,57,58] and/or may manifest from physical limitations, such as fatigue, gastrointestinal symptoms, and other physical limitations [50]. While not researched, it has been posited that these factors, along with academic performance challenges, may be both a symptom of, and contributor to, school absenteeism in PBT survivors in a self-perpetuating cycle [47].

### 2.3. Psychosocial Challenges

As described above, psychosocial difficulties can contribute to, and manifest from, school absences for PBT survivors. Irrespective of school absences, psychological and adjustment issues can independently affect school performance and engagement. PBT survivors tend to be at higher risk for depression, suicidal ideation, anxiety, and maladaptive behavior relative to the general population [59]. Further, low social acceptance, social isolation, and victimization are difficulties faced by PBT survivors [60]. Each of these factors is associated with academic difficulties, and each is potentially modifiable in a manner that can be positive and protective for the PBT survivor.

Social competence deficits are pervasive for PBT survivors [61] and represent a pain point with regard to educational success. While PBT survivors view school friendships as important and as one of the most positive aspects of their educational experience [62], feelings of isolation and rejection, along with feelings of unwanted attention and questions from peers about their brain tumor and treatment, add to stress about social competence and ability to keep up with classmates [62,63,64]. Feelings of low self-esteem and social incompetence can further lead to social inhibition, thus limiting practical peer supports in the classroom, such as help with homework or support managing difficult social circumstances at school [53].

Family functioning has been cited as protective for HRQOL in PBT survivors [65]. Specific to academic achievement, lower conflict and higher support is associated with improved achievement in PBT survivors, irrespective of cognitive risk factors [66]. Indirect evidence for family functioning as a variable associated with educational success in PBT survivors includes findings that level of family stress is associated with intellectual functioning [67] and that better psychological outcomes for children with cancer are associated with low family conflict and high cohesion and support [68]. The increased neurocognitive burden is associated with poorer family functioning [69], though the direction of this relationship is unclear. Regardless, evidence points to family functioning as an important target to potentially improve academic performance and achievement in PBT survivors.

### 2.4. Stakeholder Knowledge and Communication

Communication between parents, educators, and medical teams is essential for optimal educational support for the PBT survivor, yet researchers consistently report communication problems and gaps among all stakeholders thus representing still another significant pain point for PBT survivors. For example, parents report feeling unprepared by the oncology team regarding their child’s educational needs [70], and at the same time, healthcare teams report being unsure about how to help parents navigate the complexities of school [57] and lack knowledge of specific criteria for eligibility for special services at school [71]. In addition, even if informed by the medical team of potential treatment-related limitations, individual healthcare providers may have different assessments and methods of communication of cognitive and academic risk, and parents may misunderstand those risks [72].

Survivors and families express concern about the lack of educator knowledge in how to manage educational issues for the PBT survivor [62]. Teachers may feel ill-informed regarding the range of difficulties experienced by PBT survivors [53,73] and do not have the time, expertise, or perceived competence to manage the special needs of PBT survivors [57]. Evidence suggests this remains the case even for teachers trained in special education [74]. Lack of teacher preparation can result in parental frustration that educators seem unaware of details about their child’s condition and needs [53]. 

Challenges to optimal communication about a PBT survivor’s condition and needs among stakeholders can result in families and educators having differing views on the child’s learning and psychosocial needs, expected educational trajectory, and need for support at school [64]. Even if an educator learns more about a PBT survivor’s needs, communication gaps and lack of continuity may exist within schools such that subsequent teachers may not be informed or prepared to optimally work with a PBT survivor [75,76]. Beyond information about the needs of PBT survivors, support for educators is also lacking [77] and may be an additional barrier to optimal educational experiences for PBT survivors. Indeed, educator support has shown to have a positive impact on the classroom socialization and academic performance of PBT survivors [48]. 

### 2.5. Physical Challenges

Risks for physical disability and sensory difficulties as a result of brain tumor, treatment, and associated complications have implications for school performance and participation. Due to persisting difficulties with balance and coordination, PBT survivors are at risk for compromised mobility and poor cardiopulmonary fitness [78] which may impact school participation and engagement [53]. Fine motor control may be compromised, thus limiting the efficient performance of functional classroom activities [79]. Ototoxicity secondary to chemotherapy treatment can result in hearing loss [80] and vision impairment secondary to optic neuropathy [81] may further limit PBT survivors’ full participation in school. Finally, visible appearance changes including scarring and hair loss are associated with lower self-perception compared to the general population [82], possibly further contributing to psychosocial barriers to educational performance and attainment as described above.

**Figure 1:**
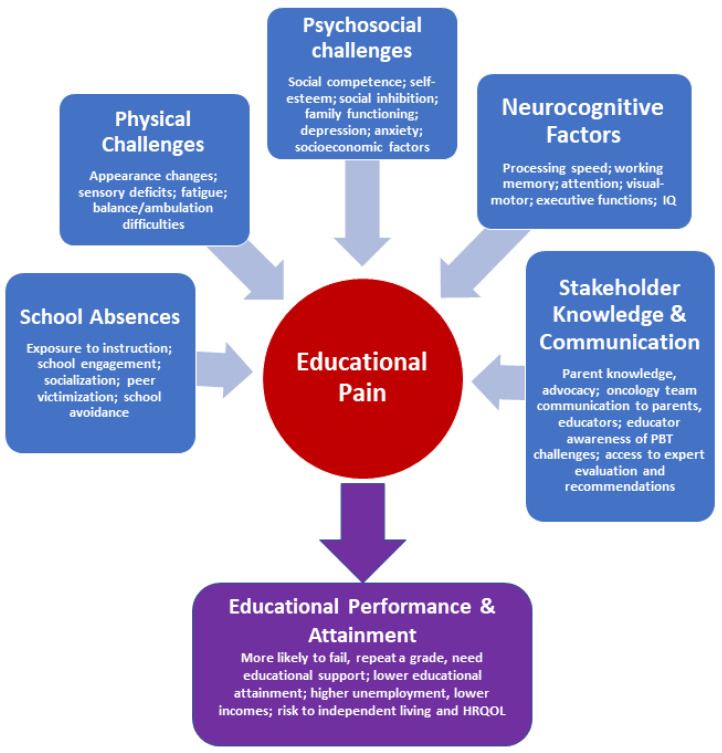
Educational Pain Points for Pediatric Brain Tumor Survivors.

Figure 1 summarizes the major educational pain points for PBT survivors.

In sum, factors ranging from neurocognitive and psychosocial changes to decreased school engagement contribute to poorer educational performance and attainment in PBT survivors. Further, support systems may not be well-coordinated or adequately knowledgeable to navigate the complexities necessary to optimize educational performance and outcomes for PBT survivors. There is likely a cumulative effect of individual factors summarized above, though research to date tends to focus on one aspect or another without investigating combinations of risks. Just as neurocognitive dysfunction progresses over time [83] for many PBT survivors, it may be that there is a compounding effect of lost opportunity, such as through limited educational engagement and decreased restorative capacity due to cognitive impact and suboptimal support that similarly increases with time. However, further investigation will be necessary to better understand the combined and cumulative impact of this group of factors over time.

Finally, the overarching impact of specific socioeconomic factors including parental education and family income cannot be ignored as factors that contribute risk to educational outcomes for PBT survivors [16]. While difficult to ameliorate at the healthcare provider or educator level, such disparities contribute to and exacerbate many of the risk factors outlined above and need to be considered when modeling risk and mitigation options for individual PBT survivors.

## 3. Educational Pain Management for Pediatric Brain Tumor Survivors

### 3.1. Targeted Intervention

At this time, there is no consensus on best practices to address educational pain points experienced by PBT survivors. Literature on intervention is piecemeal and tends to focus on discrete skills and functions. Trials purporting to improve functions related to educational performance and outcomes tend to lack inclusion of real-world metrics of educational performance and attainment. Results thus far are not yet particularly compelling to support the broad adoption of specific paradigms. 

For instance, a number of studies have focused on neurocognitive remediation and training after treatment for PBT. Online/computerized training for working memory has been shown to be acceptable and feasible with some targeted skill improvement in PBT survivors after treatment [84,85,86,87,88] though translation to meaningful changes in everyday cognitive and educational performance and outcome is lacking. A prophylactic approach using computer-based reading practice during PBT treatment did not result in significant findings [89]. While computerized training approaches certainly have appeal, the reality is that existing evidence remains lacking that these types of time-intensive programs result in meaningful, functional improvement in everyday academic performance and educational attainment.

Cognitive remediation therapies delivered in person have been evaluated in terms of improving cognitive and academic functions in PBT survivors, though like with computerized interventions, results have been generally equivocal in terms of positive impact on real-world academic performance [90,91]. A small study trained parents in behavioral modification, cognitive instructional methods, and compensatory strategies to improve ongoing intervention in the child’s natural environment. Results suggested modest benefit relative to non-intervention controls on specific academic test scores, and a correlation was noted between time spent in intervention and improvement on a reading comprehension task [92]. External incentives have shown the potential to improve situational academic performance in PBT survivors [93], suggesting that intrapersonal factors, such as level of intrinsic achievement motivation and responsivity to situational incentive may be fruitful areas of future research.

Methods for ameliorating school absences and subsequent multidimensional educational and psychosocial consequences have not been the focus of intervention trials thus far. While psychological interventions have demonstrated efficacy in relieving behavioral and emotional difficulties experienced by PBT survivors [94,95], educational impact and endpoints, such as school avoidance or classroom performance, are typically not included in trials so the impact of such interventions on educational performance and attainment are unclear.

Stakeholder knowledge may be enhanced if PBT survivors receive neuropsychological evaluation which can elucidate specific educational needs for an individual survivor while bridging the communication gap among families, educators, and medical teams [8]. Yet even though neuropsychological services are considered standard of care for PBT survivors [96], these services tend to be underutilized [8] and implementation of recommended support incomplete [97]. Further, there are practical obstacles to accessing neuropsychological evaluation with barriers including the availability of clinicians with neurocognitive assessment expertise as well as inconsistencies in billing and reimbursement [98] which limits patient access.

### 3.2. Hospital-School Liaison and Reentry Programs and Professionals

A positive development in addressing the educational pain points faced by PBT survivors is the evolution of structured school reentry programs and hospital educators and educational liaisons. Recently, a standard of care was developed for pediatric oncology patients that includes having a well-informed oncology team member assist with coordination of communication between child/family, educators, and the healthcare team to ensure support at school reentry and beyond [73]. Indeed, hospital based comprehensive school liaison programs are well-received and helpful to parents and educators [99,100]. Further, under the broad umbrella of hospital-school liaison programs, parents have been effectively trained to advocate for school support services [101] and educators’ knowledge, confidence, and comfort in managing difficulties experienced by pediatric cancer patients have shown to benefit [100,102]. Several studies have demonstrated improved peer knowledge and improved peer attitudes toward the child with cancer [103], along with improved academic achievement and learning and social adjustment of the PBT survivor [100,103] as a function of reentry programs and hospital-based educational intervention.

**Figure 2:**
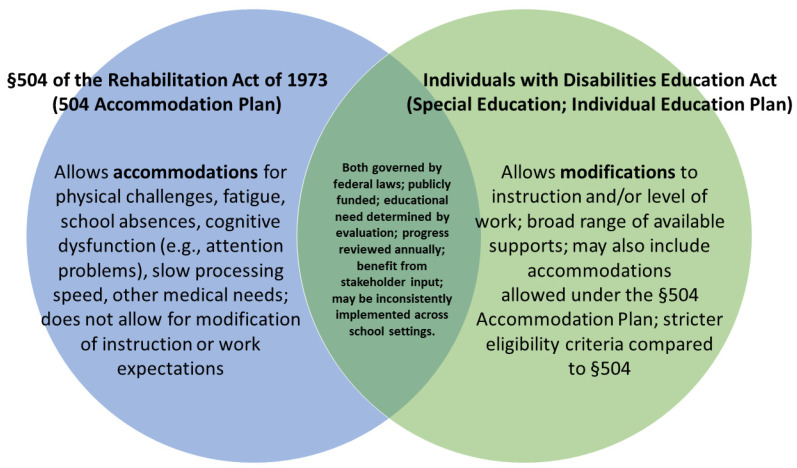
Public School Support Options for PBT Survivors with Educational Needs.

A central function of hospital-school liaison programs is to improve access to existing supports and programs available to public school students which remain the primary educational intervention for PBT survivors [104]. Figure 2 summarizes public school support options relevant to PBT survivors which include an Individual Education Plan (IEP) under the Individuals with Disabilities Education Act (IDEA) and accommodations available to individuals with a disability under Section 504 of the Rehabilitation Act of 1973. While only a minority of PBT survivors access such supports, oncology team members, such as hospital educators and liaisons, as well as allied health clinicians working with the PBT survivor, may increase access to these important supports [8] which are associated with improved educational outcomes for PBT survivors [105] but inconsistently implemented across students and schools.

While there is a paucity of research investigating essential components and best practices of hospital-school liaison programs, recommendations and guidance is emerging through organizations, such as the Hospital Educators and Academic Liaisons Association (HEAL) [106] with ongoing efforts to refine best practices and essential components of such programs [107]. The importance of ongoing research cannot be understated, particularly given that costs for such programs are often not reimbursable [70] which is partly why the standard of supporting school reentry for PBT survivors is inconsistently met. Having a strong empirical rationale for essential components and best practices may help to improve PBT survivor access to comprehensive hospital-school liaison and school support programs.

Within school settings, whether the educational needs of PBT survivors are appropriately identified and whether supports provided are optimal to PBT survivor educational performance and outcomes remains unknown [108]. Without empirical guidance, it can be difficult to identify and advocate for PBT survivor needs in school, and this may be magnified without access to a clinical provider or hospital educator with expertise in the educational needs of PBT survivors. While empirical support is lacking, in our experience there are a number of supports that are relevant to consider in the educational setting to address common issues in PBT survivors, and these are summarized in Table 1.

## 4. Opportunities for Future Research

A number of recommendations to future researchers stem from this integrated review of the literature. First, it is essential to include ecologically valid metrics of real-world academic success in intervention trials that aim to improve academic performance and outcomes, even if indirectly. For example, a cognitive intervention trial may seek to improve a discrete skill, but the real value of the intervention is in its ability to improve real-world performance. Valid endpoints may include objective metrics like school attendance, grades, level of support, and state-mandated standardized test performance. Collecting school-based performance information pre and post intervention may be useful in providing both quantitative and qualitative changes in educational metrics and should incorporate the level of support received by the PBT survivor so that performance can be understood within that context.

While the emergence of school reentry and hospital-school liaison programs is certainly positive, there is a lack of empirical support for essential elements and best practices. These types of supports can positively affect multiple endpoints (e.g., educator knowledge and competence, parental stress and informed advocacy for their child, improved peer knowledge and support, among other potential benefits.), all of which can support and optimize the PBT survivor’s academic performance and outcome. Further, hospital educators and liaisons are uniquely positioned to contribute to integrated interventions used before, during, and after treatment aimed at improving educational outcomes. Finally, a better understanding of home/school/medical communication methods, content and timelines can help right-size hospital resource utilization while optimizing positive educational impact.

Methods to decrease school absenteeism are vastly understudied in the PBT survivor population yet may be amenable to practical modifications. As discussed above, absenteeism manifests both from a practical need to attend medical appointments and treatment but also potentially from factors related to school avoidance and psychosocial vulnerability. These factors highlight the importance of practical changes, such as efforts to prioritize medical appointments outside of school hours, as well as to better engage educators in recognizing and supporting PBT survivors who may show signs of school avoidance. Similarly, while family functioning is a known risk factor for PBT survivor educational performance, there are virtually no intervention studies examining ways to improve family functioning and subsequent PBT survivor educational performance and outcome. Similarly, factors such as SES and other intraindividual factors, such as intrinsic motivation and achievement orientation are ripe for further study to potentially mitigate risk and improve classroom performance and academic outcomes for PBT survivors.

Integrating educational experts into PBT survivor intervention research is also recommended. For example, hospital educators and liaisons are viewed positively by stakeholders, and perhaps an analogous framework could be implemented and tested on the education side. Specifically, an educator could be identified who takes responsibility for in-depth learning about educational risks and manifestations secondary to PBT to facilitate support access and educational service delivery. Further, including experts in curriculum and instruction in PBT intervention trials may help identify educator methods and techniques that can improve PBT survivor performance. This can facilitate both the development of innovative education methods and optimize the use of existing supports.

Educational supports and interventions outside of school have received little attention in the PBT survivor literature. For example, within school settings, peer tutoring can be effective in improving aspects of academic performance in students with special needs [109], but tutoring outside of school has not been explored in relation to PBT survivors. Technology resources, such as consistent internet access have not been adequately explored as supports for educational performance and progress for PBT survivors. Yet reliance on supports outside of school has potential limitations. Difficulties with processing speed and cognitive stamina may complicate the potential utility of outside-of-school interventions, and future researchers may need to consider ways to circumvent these common late effects when considering interventions that are in addition to typical school demands. A broad goal of such research should be to develop an evidence base for effective supplemental supports for PBT survivors, as this can inform policy to mitigate socioeconomic inequalities that are already known to impact outcomes for PBT survivors and family access to potentially effective resources and education [16].

Finally, the complexity and multidirectional nature of risk factors for poor educational performance and outcomes for PBT survivors are rarely represented in intervention research. There is practically no research on the interplay between various risk factors, and similarly no research investigating the cumulative effects of risk factors for PBT survivor educational performance and outcome. As discussed above, each risk factor affects others (e.g., absenteeism may affect psychosocial functioning which affects academic performance which then contributes back to absenteeism; cognitive performance may affect social competence which affects academic performance). Full recognition of the risk factors to educational performance and outcomes for PBT survivors, and representation of these in intervention trials to the extent that is feasible, may significantly improve our understanding of the constellation of factors most prominently affecting performance, the interplay and cumulative effects of these over time, and best practices for remediation and management.

## Figures and Tables

**Table 1:** Examples of Educational Supports for PBT Survivors.

Common Educational Supports for PBT Survivors
**Academic Needs**
▪ Identify oncology team member (e.g., hospital educator/liaison, psychosocial team member) to communicate medical and academic needs to educators ▪ Utilize Homebound, virtual, and/or hospital-based academic support options to maintain academic continuity while student is out of school; consider flexible attendance options (partial day attendance, intermittent Homebound) ▪ Implement 504 Accommodation Plan and/or Individual Education Plan as indicated by student needs, and update plans often particularly during initial adjustment back to school ▪ Anticipate an adjustment period when student transitions back to school (e.g., expect physical and cognitive fatigue, emotional and social adjustment issues) ▪ Provide curriculum-based and classroom-based assessment to determine academic levels and workload tolerance as child adjusts back to school ▪ Start classroom accommodations upon return with no waiting–avoid failure or frustration; revise/modify accommodations and wean as appropriate: ➢ Reduce academic expectations initially, including reduced workload and homework ➢ Adjust workload to emphasize quality over quantity ➢ Communicate with parents to assess student fatigue and capacity to complete homework; communicate fatigue to educators ➢ Provide extra time to complete work or eliminate timing requirements ➢ Offer small group instruction with additional opportunities for explanation and re-teaching especially during adjustment back to school ➢ Monitor student’s adjustment/adaptation and gradually increase demands ➢ Provide organizational strategies (visual checklists, routines, step-by-step procedures, help with planning / organizing) ➢ Provide memory strategies (auditory, visual, tactile modalities) ➢ Utilize technology to preserve energy (e.g., text-to-voice, voice-to-text, calculator, etc.) ➢ Consider altered testing formats (oral testing, multiple choice formats, open note tests)
**Psychosocial Needs**
▪ With parent and student permission as appropriate, communicate with medical team to see if team member can educate class on child’s condition and needs (e.g., hospital educator, nurse, Child Life Specialist) ▪ Offer staff development/in-service to promote staff awareness of child’s condition and dispel assumptions and anxieties about PBT survivor (e.g., not contagious, etc.) ▪ Allow student to wear hat / scarf for hair loss–encourage peers to do so as well ▪ Offer student opportunity to tell her/his story to the class (e.g., oral presentation, photographs/video) ▪ Respect individual preferences–some students may not wish to talk about their condition or experience and may prefer little or no special attention ▪ Consider counseling in school from guidance counselor or school psychologist ▪ Offer opportunities for peer support as needed (e.g., friendship group, lunch buddies, peer partners) ▪ To the extent possible, normalize academic and social demands placed on the child ▪ Reduce emphasis on competition within the classroom ▪ Reduce stress or time constraints ▪ Communicate regularly with family to monitor student and family stress; offer support and referrals as needed ▪ Provide student with opportunities for success and leadership to build esteem
**Physical Needs**
▪ During the initial transition, offer flexible attendance options to manage fatigue and medical appointments (e.g., late start or early release from school to shorten, use electronic communication to facilitate instruction and assignment completion) ▪ Consider early release from class to avoid crowds in hallways, extra time between classes ▪ Provide breaks / rest time if needed ▪ Assess independent ambulation and offer accommodations to ensure student safety ▪ Preferential locker access to reduce walking/carrying ▪ Peer buddy to carry books and materials ▪ Consider set of textbooks at home to reduce carrying books back and forth between home and school ▪ Avoid stairs, allow elevator access as needed ▪ Allow restroom breaks as needed by student ▪ Water bottle, snacks allowed for hydration and energy as directed by medical team ▪ Communicate with medical team regarding participation in physical education and sports ▪ Maintain communication with family over time to ensure educational implications of medical status are known to educators; maintain communication with medical team as appropriate
